# Case Report: Pancreatic sclerosing epithelioid mesenchymal neoplasm (SEMN) with novel pancreatic duct obstruction in a middle-aged female

**DOI:** 10.3389/fmed.2026.1782497

**Published:** 2026-04-10

**Authors:** Zhiwei Wang, Yifan Liu, Haowei Feng, Shizhen Zhang, Longfei Dai, Xianping Cui

**Affiliations:** Department of Hepatobiliary Surgery I, Shandong First Medical University Affiliated Shandong Provincial Hospital, Jinan, Shandong, China

**Keywords:** case report, laparoscopic spleen-preserving distal pancreatectomy (SPDP), pancreatic duct obstruction, pancreatic sclerosing epithelioid mesenchymal neoplasm (SEMN), pancreatic neuroendocrine tumor (pNET)

## Abstract

Pancreatic sclerosing epithelioid mesenchymal neoplasms (SEMN) are extremely rare mesenchymal tumors, with only approximately 10 cases reported globally to date, resulting in limited understanding of their clinical, imaging, and pathological characteristics. We present a 55-year-old female admitted to our hospital following the incidental detection of a pancreatic mass during a routine health check-up. The patient had no specific clinical symptoms or relevant medical history. Preoperative multimodal imaging—including computed tomography (CT), dynamic contrast-enhanced MRI (dynamic contrast MRI), and positron emission tomography (PET)/CT—revealed a 1.6 × 1.4 cm solid lesion at the pancreatic body-neck junction, with a novel, previously unreported radiological feature: main pancreatic duct (MPD) interruption and distal dilation. Combined with postoperative histopathological findings of direct tumor infiltration into the MPD wall and disruption of ductal epithelium, this confirmed true ductal invasion rather than mechanical compression. Due to overlapping imaging manifestations with common pancreatic tumors, initial differential diagnosis strongly favored pancreatic neuroendocrine tumor (pNET). A preoperative attempt at duodenoscopic pancreatic duct stenting was performed to restore ductal patency and facilitate organ-sparing resection; however, the procedure failed, presumably due to tumor infiltration of the MPD. Intraoperative frozen section analysis was unable to definitively characterize the lesion, prompting laparoscopic spleen-preserving distal pancreatectomy (SPDP) to ensure complete resection and rule out malignancy. Postoperative histopathology demonstrated nests of epithelioid and spindle cells embedded within a dense sclerotic stroma, and immunohistochemical staining (Vimentin+, CK8/18+, CD99+; Syn–, CgA–, INSM1–) confirmed the diagnosis of SEMN. The patient had an uneventful postoperative recovery, with no evidence of recurrence or metastasis at the one-year follow-up. This case expands the clinical and imaging spectrum of SEMN by documenting its occurrence in middle-aged females and identifying MPD obstruction as a novel radiological phenotype. It further highlights the diagnostic pitfalls of SEMN due to imaging overlap with prevalent pancreatic tumors, and provides valuable insights for preoperative differential diagnosis and individualized surgical management—particularly the necessity of adapting resection strategies when minimally invasive ductal interventions fail to enable organ-sparing procedures. Collectively, our findings underscore the importance of integrating preoperative imaging, interventional outcomes, intraoperative observations, and postoperative immunohistochemical results to avoid misdiagnosis of this rare entity.

## 1 Introduction

Pancreatic mesenchymal tumors account for merely 1–2% of all pancreatic neoplasms, arising predominantly from connective, lymphoid, vascular, or neural tissues (1). Within this rare subgroup, sclerosing epithelioid mesenchymal neoplasm (SEMN) represents an ultra-rare entity, first formally characterized by Basturk et al. (2) in 2019. As of December 2025, only approximately 10 cases have been documented worldwide, leading to a paucity of evidence regarding its pathogenesis, clinical manifestations, and optimal management strategies.

SEMN predominantly affects young to middle-aged females, with most lesions localized to the pancreatic head and neck (2, 3). Clinically, it typically presents as a solitary, solid, well-demarcated nodule lacking a capsule, pathologically defined by alternating nests of epithelioid and spindle cells embedded within a dense sclerotic stroma (3). Owing to its extreme rarity and absence of specific clinical symptoms, SEMN is often incidentally detected during routine health check-upor imaging studies performed for unrelated indications. Its imaging features—including hypodense signals on computed tomography (CT), isointense T2 signals on dynamic contrast MRI, and progressive mild enhancement—frequently overlap with those of more common pancreatic tumors, particularly pancreatic neuroendocrine tumors (pNETs) and solid pseudopapillary neoplasms (SPNs) (4). This substantial imaging overlap poses formidable diagnostic challenges, resulting in a high rate of preoperative misdiagnosis, with definitive diagnosis typically reliant on postoperative histopathological and immunohistochemical confirmation.

Notably, three critical knowledge gaps persist in the current understanding of SEMN. First, pancreatic duct obstruction—a radiological feature commonly associated with malignant pancreatic lesions or high-grade pNETs (5)—has never been documented in SEMN prior to this study, limiting the comprehensiveness of its imaging spectrum. Second, the utility of preoperative minimally invasive interventions (e.g., duodenoscopic pancreatic duct stenting) to facilitate organ-sparing surgery remains entirely unexplored, as no prior cases have attempted such therapeutic approaches. Third, the malignant potential of SEMN remains controversial: while most reported cases exhibit an indolent clinical course, duct invasion—an established indicator of aggressiveness in similar rare mesenchymal tumors (6)—has not been verified in SEMN, leaving its biological behavior incompletely elucidated.

Against this backdrop, we report a case of incidentally detected SEMN located at the pancreatic body-neck junction in a 55-year-old female, which presented with the novel radiological feature of MPD interruption and distal dilation. We detail the stepwise diagnostic workflow, including the failed preoperative duodenoscopic stenting attempt, subsequent minimally invasive surgical resection, and confirmatory histopathological findings. This case expands the clinical and imaging spectrum of SEMN, enhances clinicians' awareness of its atypical presentations, and provides evidence-based insights for preoperative differential diagnosis and individualized management of this underrecognized disease.

## 2 Case presentation

A 55-year-old female was incidentally found to have a pancreatic mass during a routine health check-up in November 2024, prompting her admission to our hospital for further assessment. She had no relevant medical history, including chronic conditions such as diabetes mellitus, hypertension, or cardiovascular disorders, nor did she have a history of long-term medication use. She denied experiencing gastrointestinal symptoms like abdominal pain, bloating, jaundice, or unintended weight loss, and there was no family history of pancreatic tumors, inflammatory bowel disease, or other hereditary disorders.

Based on the patient's asymptomatic presentation combined with initial imaging features, the following differential diagnoses were initially considered (focusing on the most clinically relevant and easily confused lesions): 1) Pancreatic neuroendocrine tumor (pNET): Most functional pNETs present with specific clinical symptoms (e.g., insulinomas with recurrent hypoglycemia, fatigue and confusion; gastrinomas with intractable peptic ulcers, epigastric pain and gastrointestinal bleeding), while non-functional pNETs are often asymptomatic and incidentally discovered during routine health check-ups, which overlaps with the presentation of our case; the differential diagnosis was further confirmed by subsequent immunohistochemical staining (negative for Syn, CgA, INSM1, and ruling out pNET). 2) Pancreatic adenocarcinoma: Typically presents with progressive epigastric pain, unexplained weight loss, jaundice (when involving the common bile duct), anorexia and fatigue, all of which were absent in our patient, helping to preliminarily exclude this diagnosis. 3) Solid pseudopapillary tumor of the pancreas: More common in young women (predominantly 20–40 years old), most patients are asymptomatic or present with mild, intermittent epigastric discomfort, and no obvious specific clinical symptoms were found in our patient; its imaging features were further distinguished from our case.

**Figure 1:**
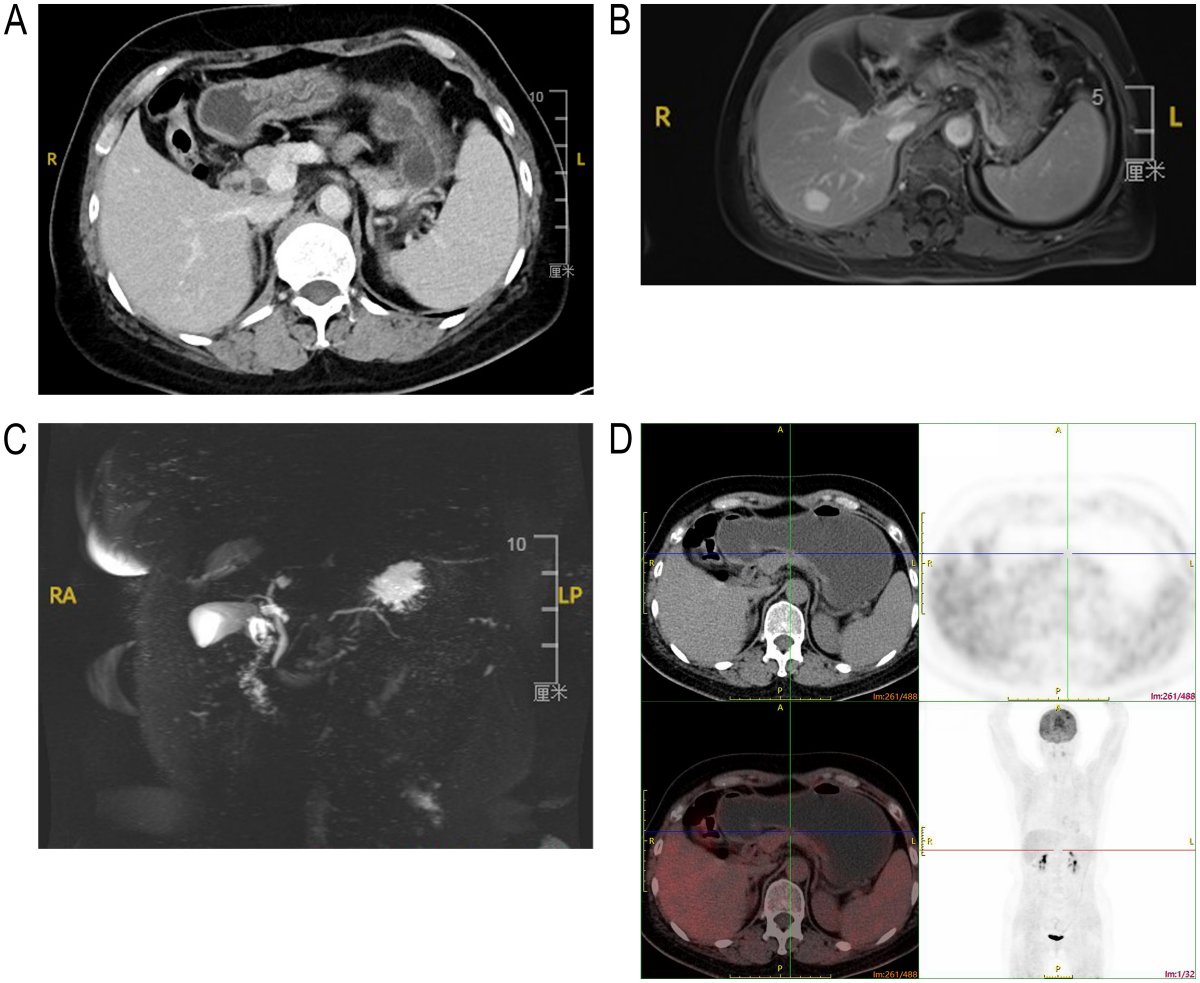
Preoperative imaging findings of pancreatic lesions. **(A)** CT revealed a solid lesion in the pancreatic body-neck junction, with ill-defined margins and no evidence of calcification or cystic degeneration. **(B)** Heterogeneous progressive mild enhancement was noted across delayed phases, with the lesion demonstrating higher signal intensity than the adjacent normal pancreatic parenchyma. **(C)** Magnetic resonance cholangiopancreatography (MRCP) revealing interruption of the pancreatic duct at the lesion site with mild dilation of the distal pancreatic duct. **(D)** PET/CT demonstrating no increased fluorodeoxyglucose (FDG) metabolism and no enhanced radioactive tracer uptake in the lesion.

Comprehensive preoperative imaging was conducted to characterize the lesion in detail. Computed tomography (CT) revealed a hypodense solid lesion at the pancreatic body-neck junction, with ill-defined margins and no signs of calcification or cystic degeneration (Figure 1A). Subsequent dynamic contrast MRI further clarified the lesion as a 1.6 × 1.4 cm solid nodule, presenting with slightly prolonged T1 signal, isointense T2 signal, and heterogeneous progressive mild enhancement across arterial, portal, and delayed phases. Notably, the lesion exhibited higher signal intensity than the adjacent normal pancreatic parenchyma in the portal and delayed phases (Figure 1B). Mild hyperintensity was observed on diffusion-weighted imaging (DWI), with corresponding hypointensity on the apparent diffusion coefficient (ADC) map. Of particular importance, dynamic contrast MRI identified a previously unreported radiological feature: interruption of the main pancreatic duct accompanied by distal dilation (Figure 1C). Despite sequential evaluation by CT and dynamic contrast MRI confirming the pancreatic neck lesion with duct obstruction, its benign or malignant nature remained unclear preoperatively. Therefore, positron emission tomography (PET)/CT was performed as a supplementary imaging modality to assess lesion metabolic activity for distinguishing benign from malignant lesions and to rule out occult lymph node or distant metastasis. The PET/CT results showed no increased fluorodeoxyglucose (FDG) uptake in the lesion, as well as no evidence of regional lymphadenopathy or distant metastases (Figure 1D), which provided crucial guidance for subsequent treatment decision-making. Laboratory tests, including liver function assessments, fasting blood glucose, serum amylase and serum tumor markers (CA19-9, CA125, carcinoembryonic antigen [CEA]), all fell within normal reference ranges (Table 1).

**Table 1:** Preoperative laboratory data.

Characteristics	Value	Unit	Reference range
ALT	30	IU/L	5–40
AST	21	IU/L	8–40
TBil	12.67	umol/L	3.42–23.34
DBil	1.99	umol/L	0–8.24
Albumin	40.1	g/L	35–55
Glucose	5.20	mmol/L	3.9–6.1
Lipase	39.81	U/L	0–160
Amylase	25	U/L	25–125
Pancreatic amylase	<20	U/L	0–50 U/L
CA 19-9	6.35	U/ml	0.01–37
CA125	6.38	U/ml	0–35
CEA	0.49	ng/ml	0.01–5

ALT, Alanine Aminotransferase; AST, Aspartate Aminotransferase; TBil, Total Bilirubin; DBil, Direct Bilirubin; CA 19-9, Carbohydrate Antigen 19-9; CA125, Carbohydrate Antigen 125; CEA, Carcinoembryonic Antigen.

Based on the imaging findings (solid lesion with mild progressive enhancement and no FDG uptake on PET/CT), the initial differential diagnosis strongly pointed to a low-grade pancreatic neuroendocrine tumor (pNET). Low-grade pNETs are characterized by indolent biological behavior, and preoperative nasopancreatic stenting has been reported to facilitate safe organ-sparing resection for small pNETs adjacent to the main pancreatic duct without confirmed infiltration (7), which may reduce the risk of duct injury and postoperative complications. Guided by this clinical evidence for pNETs with ductal adjacency rather than infiltration, an attempt at endoscopic pancreatic duct stenting via duodenoscopy was made to restore ductal patency and preserve pancreatic parenchyma in the present case. This intervention was ultimately unsuccessful, as histologically confirmed tumor infiltration of the main pancreatic duct precluded effective stent placement, a finding that aligns with the inherent limitations of minimally invasive ductal interventions in the setting of confirmed main pancreatic duct (MPD) involvement.

**Figure 2:**
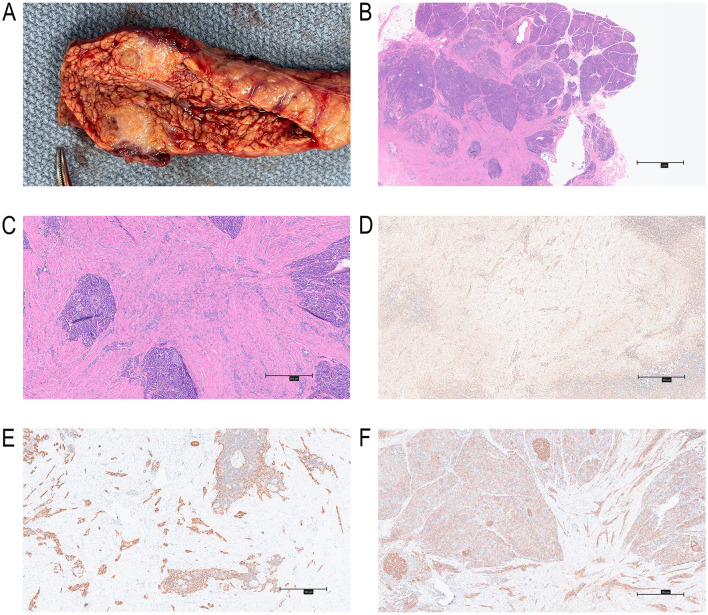
Postoperative resected specimen. **(A)**The main pancreatic duct was transversely obstructed by the tumor. **(B)** HE staining of 12.5× magnification. **(C)** HE staining of 50× magnification, histopathological sections showing a geographic pattern of the tumor, with enmeshed tumor cells forming a slit-like structure. **(D)** Vimentin (+) immunohistochemistry result. **(E)** CK 8/18 (+) immunohistochemistry result. **(F)** CD99 (+) immunohistochemistry result.

Following a multidisciplinary team (MDT) discussion, laparoscopic spleen-preserving distal pancreatectomy (SPDP) was performed in November 2024 to ensure complete resection of the lesion and rule out malignancy. Intraoperative exploration revealed no ascites, peritoneal metastases, or intra-abdominal adhesions; subsequently, extensive exploration of the abdominal cavity and peripancreatic region was further performed, showing no invasion of the spleen, adjacent visceral organs (stomach, duodenum) or blood vessels (splenic artery and vein), as well as no suspicious metastatic lymph nodes in the peripancreatic, perigastric, or splenic hilar regions. The pancreatic lesion was localized to the body-neck junction, consistent with the preoperative imaging findings, and appeared as a solid nodule with a size of approximately 1.6 × 1.4 cm, clear boundaries, and moderate blood supply, without obvious hemorrhage, necrosis, or cystic degeneration. After resecting the pancreatic body and tail specimen containing the lesion, the lesion was further explored by intraoperative incision on the operating table: it had a firm texture and a pale gray color, and visible invasion and interruption of the main pancreatic duct in the tumor area with mild dilation of the distal pancreatic duct were observed, which was consistent with the preoperative dynamic contrast MRI findings. The surgery involved en bloc resection of the tumor, distal pancreatic segment (including the capsule), and surrounding mesentery (Figure 2A), with careful preservation of the splenic artery and vein to maintain splenic perfusion and function. The total duration of the operation was 2.5 h, with an intraoperative blood loss of 40 ml. Intraoperative frozen section pathology examination was performed on the resected specimen, but the results were unable to definitively determine the nature of the lesion, only suggesting a mesenchymal tumor with uncertain malignant potential; subsequently, after communication with the patient's family, we continued the spleen-preserving distal pancreatectomy.

Postoperative histopathological examination showed an unencapsulated tumor composed of alternating nests of epithelioid and spindle cells embedded in a dense sclerotic stroma. Serial histological sections demonstrated direct infiltration of tumor cells into the wall of the main pancreatic duct (MPD), with disruption of the normal ductal epithelial layer and replacement of the underlying connective tissue by sclerotic tumor stroma. No clear boundary was identified between the tumor and the MPD wall, excluding the possibility of simple external compression. Epithelioid cells had sparse cytoplasm, round-to-ovoid nuclei, and open chromatin, while spindle cells exhibited irregular morphology and hyperchromatic nuclei. Rare mitotic figures were detected, with no evidence of vascular invasion or lymph node involvement (Figures 2B, C). Immunohistochemical staining revealed positive expression of Vimentin (+), CK8/18 (+), CD99 (+), CK(AE1/AE3)(+), AAT (+), and SSTR2 (+), and negative expression of Syn (–), CgA (–), INSM1 (–), CD56 (–), β-Catenin (–), CD31 (–), CD34 (–), ERG(–), TFE3(–), Calretinin(–), WT-1(–), D2-40(–), SMA(–), SS18-SSX(–), and Ki-67 (5% labeling index) (Figures 2D–F). These findings were consistent with a diagnosis of SEMN.

Postoperatively, the patient received anti-infective therapy with cefoperazone sulbactam (Sulperazon) at a dose of 3 g every 12 h for 1 week, along with somatostatin at 3 mg daily for enzyme inhibition until discharge. The patient achieved an uneventful postoperative recovery: flatus was passed and a liquid diet was initiated on postoperative day 5, with satisfactory tolerance to dietary advancement, and the patient was discharged on postoperative day 10. To date, no international medical society has issued a specific standard follow-up protocol for sclerosing epithelioid mesenchymal neoplasm (SEMN), which is primarily attributed to its rarity and limited accumulation of clinical cases. Therefore, the patient was advised to undergo dietary modification, regular clinical follow-up visits, and surveillance examinations—including contrast-enhanced computed tomography (CECT) for the 6-month postoperative assessment and dynamic contrast-enhanced MRI for subsequent regular surveillance, combined with serum tumor marker (CA19-9, CEA) detection—every 6 months for the first 2 years, followed by annual surveillance thereafter. This intensified follow-up protocol was formulated by combining the novel MPD invasion feature and 5% Ki-67 proliferation index of the SEMN lesion with the follow-up principles for low-grade or potentially malignant pancreatic tumors from relevant retrospective studies, to closely monitor potential tumor recurrence or progression. CECT was selected for the early postoperative follow-up to leverage its superior spatial resolution for the comprehensive evaluation of surgical resection margins, splenic vascular patency and abdominal soft tissue integrity, enabling a precise initial assessment of surgical outcomes and early recurrence risk. Dynamic contrast-enhanced MRI was designated as the long-term surveillance modality to minimize cumulative ionizing radiation exposure for the patient during prolonged monitoring, while maintaining high soft tissue contrast for the detection of subtle recurrent lesions. This follow-up strategy was formulated in accordance with the follow-up principles for low-grade malignant or potentially malignant pancreatic tumors proposed in relevant retrospective studies, which is clinically feasible and rational.

**Figure 3:**
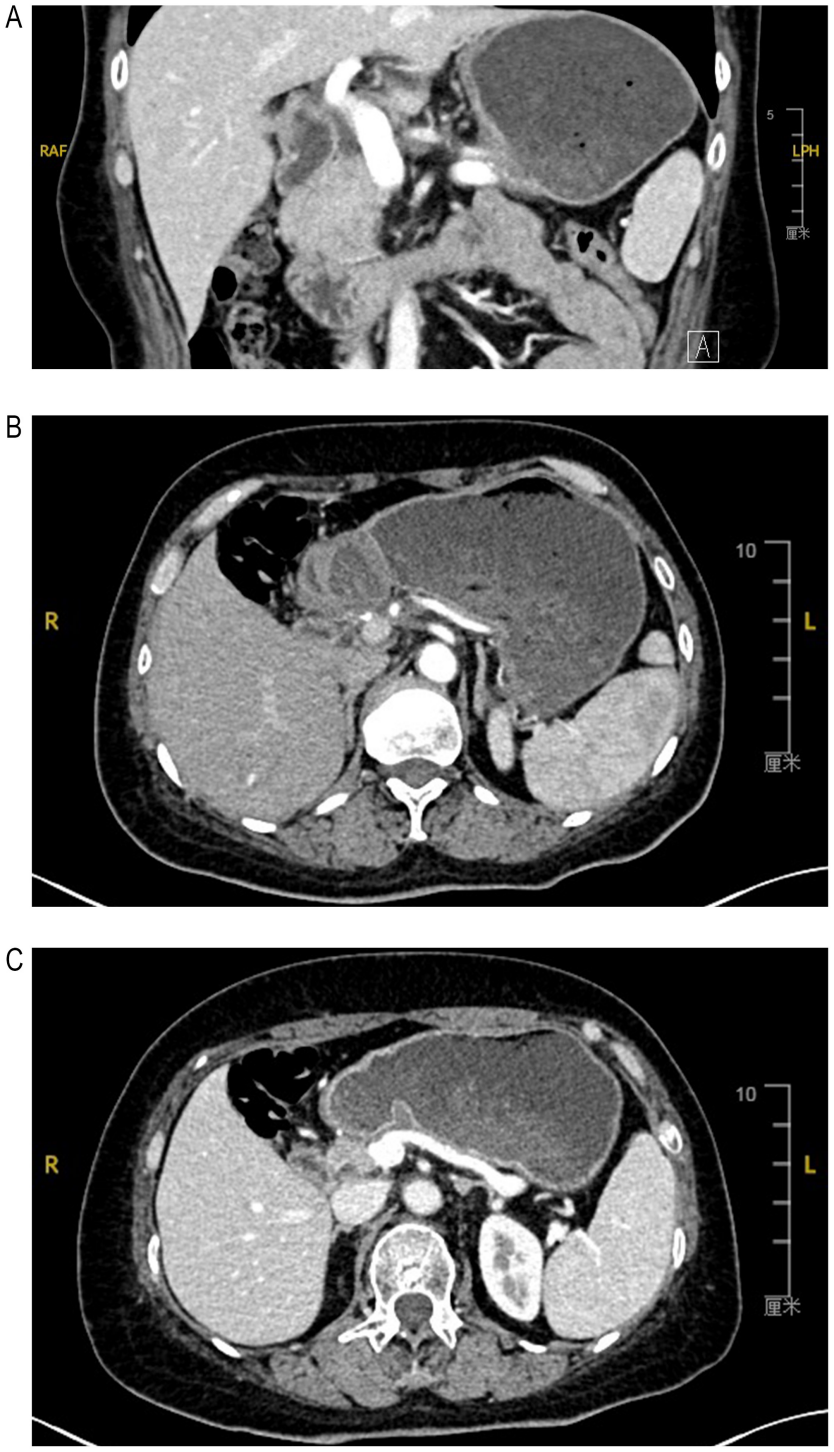
Surveillance imaging performed at 6 months postoperatively (CT). **(A)** The imaging confirmed complete tumor resection, with no evidence of local recurrence or distant metastasis. **(B, C)** The imaging demonstrated patent splenic artery and vein with well-maintained splenic perfusion.

The patient strictly followed the follow-up plan. Surveillance CT performed in May 2025 (6 months postoperatively) confirmed complete tumor resection, with no signs of local recurrence or distant metastasis (Figure 3A), and showed patent splenic artery and vein with well-maintained splenic perfusion (Figures 3B, C). At the 1-year follow-up, the patient remained asymptomatic, with persistently normal laboratory test results and no radiological evidence of tumor recurrence or metastasis. Throughout the postoperative period, she experienced no complications such as pancreatic fistula or dyspepsia, tolerated the treatment well, and maintained a good quality of life.

## 3 Discussion

Pancreatic mesenchymal tumors account for merely 1–2% of all pancreatic neoplasms, originating primarily from connective, lymphoid, vascular, or neural tissue. Among these rare lesions, sclerosing epithelioid mesenchymal neoplasm (SEMN) is an exceptionally uncommon entity first formally described in 2019 (2), with only approximately 10 cases reported globally as of December 2025 (Table S1). This paucity of clinical data results in limited understanding of its pathogenesis, biological behavior, and optimal management strategies. Herein, we report a 55-year-old female with incidentally detected SEMN at the pancreatic body-neck junction, presenting with the novel radiological feature of main pancreatic duct interruption and distal dilation—previously unreported in SEMN. Following a failed preoperative duodenoscopic pancreatic duct stenting attempt, the patient underwent successful laparoscopic spleen-preserving distal pancreatectomy (SPDP) and achieved disease-free survival at the 1-year follow-up. This case not only expands the imaging and clinical spectrum of SEMN but also provides critical insights into its differential diagnosis, biological behavior, and individualized therapeutic strategies.

A key finding of this case is the identification of histologically confirmed main pancreatic duct (MPD) invasion pancreatic duct invasion by SEMN, manifested as main pancreatic duct interruption and distal dilation. Prior to this report, pancreatic duct obstruction was considered a radiological feature associated with malignant pancreatic lesions or high-grade pancreatic neuroendocrine tumors (pNETs) (1), but never documented in SEMN. This observation raises important questions about the biological behavior of SEMN: while most previously reported cases exhibited an indolent clinical course, duct invasion—an indicator of aggressiveness in similar rare mesenchymal tumors (6)— may imply potential aggressive tendencies in individual SEMN cases. Notably, compared with previous reports describing SEMN as a benign lesion, the 5% Ki-67 proliferation index of the present lesion represents a notable finding. This index may reflect the biological heterogeneity of SEMN associated with MPD invasion, suggesting a mild elevation of proliferative activity in lesions with ductal infiltration. Based on this, an intensified follow-up protocol was implemented for the patient at our center. Future multicenter studies should incorporate the Ki-67 index into the characterization of SEMN to clarify its correlation with tumor aggressiveness and prognosis. However, given the limited number of reported cases and the 1-year follow-up duration of our patient, definitive conclusions regarding the latent malignant potential of SEMN cannot be drawn. This finding emphasizes the need for heightened clinical vigilance, as lesions involving the main pancreatic duct may require complete tumor resection as a precautionary measure to prevent potential recurrence or progression, pending accumulation of more long-term clinical data.

Additionally, this case highlights the inherent technical limitations of preoperative minimally invasive interventions for SEMN lesions with confirmed MPD infiltration. The preoperative attempt at pancreatic duct stenting in this case was guided by the initial misdiagnosis of low-grade pNET, a strategy described for pNETs confined to the periductal region without overt ductal infiltration; no clinical evidence supports the application of this minimally invasive approach in pancreatic neoplasms with preoperatively identified MPD infiltration. The failure of this intervention—attributable to histologically confirmed tumor infiltration of the MPD—reinforces that minimally invasive ductal stenting and conventional organ-sparing surgical approaches have limited applicability in the setting of MPD involvement, regardless of tumor subtype. Our multidisciplinary team (MDT) rigorously evaluated the feasibility of assessing the main pancreatic duct (MPD) using the SpyGlass direct visualization system but ultimately decided against its implementation. Preoperative duodenoscopic pancreatic duct stenting had failed, and imaging had confirmed MPD transection; this examination would not provide additional diagnostic value. Furthermore, tumor infiltration-induced MPD wall stiffening and luminal stenosis would render SpyGlass manipulation technically highly challenging, increase the risk of endoscopic procedure-related complications such as pancreatic duct perforation and acute pancreatitis, and fail to improve clinical outcomes. Therefore, our MDT formulated a laparoscopic resection regimen for the patient. For SEMN lesions with confirmed MPD infiltration, such findings indicate that organ-sparing strategies tailored for pNETs with ductal adjacency are not clinically applicable, and preoperative surgical planning should prioritize complete tumor resection to ensure oncological adequacy, rather than attempting organ-preserving interventions that are unlikely to be technically feasible.

SEMN lacks specific clinical symptoms and distinctive radiological hallmarks, leading to frequent overlapping imaging manifestations with common pancreatic tumors—particularly pNETs, solid pseudopapillary neoplasms (SPNs), sarcomatoid undifferentiated carcinoma (SUC), and sclerosing epithelioid fibrosarcoma (SEF). Accurate differential diagnosis is critical to avoid mismanagement, and core distinguishing features are summarized below:

### 3.1 Pancreatic neuroendocrine tumors (pNETs)

Pancreatic neuroendocrine tumors (pNETs) represent the most critical differential diagnosis for SEMN, primarily due to significant overlap in their imaging manifestations (8). Specifically, low-grade pNETs (G1) typically exhibit high signal intensity on dynamic contrast MRI contrast-enhanced arterial and portal phases (9), whereas high-grade pNETs (G2/G3) present with low or isointense signals on portal and delayed phases, restricted diffusion on diffusion-weighted imaging (DWI), and main pancreatic duct dilatation (10)—a radiological feature that our SEMN case also shared. However, immunohistochemical staining serves as the definitive basis for differentiating these two entities: the 2022 WHO Classification of Neuroendocrine Neoplasms (11) clearly defines that pNETs consistently express the core neuroendocrine markers synaptophysin (Syn), chromogranin A (CgA), and insulinoma-associated protein 1 (INSM1), whereas our SEMN case was negative for all these neuroendocrine markers and positive for cytokeratin 8/18 (CK8/18), cluster of differentiation 99 (CD99), and vimentin. Therefore, the exclusion of pNETs (12) in our case was primarily supported by two key pieces of evidence: the negative immunohistochemical staining for the core neuroendocrine markers (Syn^−^, CgA^−^, INSM1^−^) and the distinctive histopathological features of SEMN, which are consistent with the diagnostic criteria established by the 2022 ENETS Consensus Guidelines for the Diagnosis and Management of Pancreatic Neuroendocrine Neoplasms (13), further validating the rigor of our differential diagnosis.

### 3.2 Solid pseudopapillary neoplasms (SPNs)

SPNs predominantly affect young women aged 20–40 years (14), which differs from our 55-year-old patient. Imaging of SPNs typically reveals cystic-solid masses with peripheral solid components and a “floating cloud sign” (15), distinct from the pure solid lesion with pancreatic duct obstruction observed in our case. Histopathologically, SPNs are characterized by pseudopapillary structures around fibrous vascular bundles and low Ki-67 expression (16), while SEMN features epithelioid/spindle cell nests embedded in dense sclerotic stroma. Immunohistochemically, SPNs lack expression of CK8/18 and CD99—markers that were positive in our SEMN case—providing a key diagnostic distinction.

### 3.3 Sarcomatoid undifferentiated carcinoma (SUC)

SUC is a highly malignant tumor that primarily affects the elderly, presenting with solid/cystic masses and indistinct borders on imaging (17). While SUC exhibits dual epithelial (CK, EMA) and mesenchymal (Vimentin) marker expression (18)—sharing Vimentin positivity with SEMN—it is distinguished by high mitotic activity, pleomorphism, and frequent KRAS mutations (19). In contrast, our SEMN case showed rare mitotic figures, no vascular/lymphatic invasion, and negative EMA expression. Clinically, SUC has a poor prognosis with a median survival of ~13 months (20), which contrasts sharply with the indolent course observed in our patient at the 1-year follow-up.

### 3.4 Sclerosing epithelioid fibrosarcoma (SEF)

SEF is an extremely rare pancreatic lesion with uniform-density soft tissue masses and lower CT values than normal pancreatic tissue (21). Histopathologically, it shares epithelioid cells in sclerotic stroma with SEMN, but is genetically distinguished by EWSR1-CREB3L1 gene fusion (22) and weak/negative expression of CK8/18 (which was strongly positive in our case). Clinically, SEF is prone to recurrence and metastasis (23), whereas our patient showed no evidence of recurrence at follow-up, further supporting the SEMN diagnosis.

For SEMN involving the pancreatic body-neck junction, laparoscopic spleen-preserving distal pancreatectomy (SPDP) is a feasible treatment option, as demonstrated by this case. The surgery achieved complete tumor resection while preserving splenic function through careful protection of the splenic artery and vein, resulting in an uneventful postoperative recovery and no long-term complications. Given the potential malignant implication of duct invasion, complete resection is recommended for SEMN lesions, particularly those involving the main pancreatic duct.

Regarding follow-up, although SEMN exhibits an indolent behavior in most reported cases, the presence of duct invasion in our case warrants long-term surveillance. We recommend regular imaging (e.g., CT or dynamic contrast MRI) every 6 months for the first 2 years, followed by annual surveillance thereafter, to monitor for recurrence or metastasis. Laboratory tests, including tumor markers and liver/renal function assessments, should also be included in follow-up evaluations to ensure comprehensive disease monitoring.

This study has several limitations. First, as a single-case report, the findings may not be generalizable to all SEMN patients, and additional cases are needed to confirm the prevalence of pancreatic duct obstruction as an imaging feature of SEMN. Second, molecular genetic analyses were not performed, which could further validate the diagnosis and elucidate the genetic background of this rare neoplasm; this was due to the lack of institutionally validated, standardized molecular testing kits for SEMN and technical limitations in targeted genetic analysis for rare mesenchymal tumors at our institution. Third, the long-term biological behavior of SEMN—particularly the malignant potential of duct-invading lesions—remains unclear due to the limited follow-up duration (1 year) and small number of reported cases. The indolent course observed in our patient is consistent with most published reports, but the clinical significance of duct invasion in SEMN cannot be generalized and requires validation in larger cohorts with extended follow-up periods. Longer follow-up of this patient, accumulation of more clinical data, and complementary molecular analyses in subsequent research will be critical to further clarify the natural history and genetic characteristics of SEMN.

In conclusion, this case identifies pancreatic duct interruption with distal dilation as a novel imaging feature of SEMN, expands the known clinical spectrum of the disease, and provides practical insights for differential diagnosis and management—including the important observation that preoperative duodenoscopic pancreatic duct stenting may be technically unfeasible in SEMN lesions invading the main pancreatic duct due to tumor infiltration. The integration of preoperative imaging, interventional findings (such as the outcome of stenting attempts), intraoperative observations, and postoperative immunohistochemical results is essential to avoid misdiagnosis of this rare entity. Individualized surgical strategies—adapted based on lesion location, duct involvement, and preoperative intervention outcomes—are key to optimizing patient outcomes. Further research and multi-center case series are needed to refine our understanding of SEMN's biological behavior and establish evidence-based diagnostic and therapeutic guidelines.

## 4 Patient perspective

When I was diagnosed with a pancreatic mass during a routine physical in November 2024, I felt anxious and confused—having no prior symptoms, a healthy lifestyle, and no chronic disease history, the unexpected finding left me uncertain about my condition. The medical team eased my fears by patiently explaining each diagnostic test (CT, dynamic contrast MRI, PET/CT) and their purposes, as well as the differential diagnosis and rationale for preoperative duodenoscopic pancreatic duct stenting. Even when the stenting attempt failed, they promptly adjusted to recommend laparoscopic spleen-preserving distal pancreatectomy, and their professionalism gave me great confidence in moving forward with treatment.

My recovery was smooth: I passed flatus and resumed a liquid diet by postoperative day 5, with no adverse events like pancreatic fistula or infection, and was discharged on day 10. Following the doctors' advice on diet modification and regular follow-up, 6-month and 1-year postoperative CT scans confirmed no recurrence, and my laboratory results remained normal—bringing me immense peace of mind. Today, I have returned to normal daily life and maintained a healthy lifestyle. I am deeply grateful to the entire medical team for their expertise, compassion, and personalized care, and hope my experience encourages other patients with similar rare conditions to trust their healthcare providers and feel confident in seeking treatment.

## Data Availability

The original contributions presented in the study are included in the article/Supplementary material, further inquiries can be directed to the corresponding authors.
